# A cross sectional study of anemia and iron deficiency as risk factors for arsenic-induced skin lesions in Bangladeshi women

**DOI:** 10.1186/s12889-016-2824-4

**Published:** 2016-02-16

**Authors:** Molly L. Kile, Joycelyn M. Faraj, Alayne G. Ronnenberg, Quazi Quamruzzaman, Mahmudar Rahman, Golam Mostofa, Sakila Afroz, David C. Christiani

**Affiliations:** College of Public Health and Human Sciences, Oregon State University, 15 Milam Hall, Corvallis, OR 97331 USA; Department of Nutrition, School of Public Health and Health Sciences, University of Massachusetts, Amherst, 100 Holdsworth Way, Amherst, MA 01003 USA; Dhaka Community Hospital Trust, 190/1 Baro Moghbazar, Wireless Railgate, Dhaka, Bangladesh; Department of Environmental Health, Harvard TH Chan School of Public Health, 677 Huntington Avenue, Boston, MA USA

**Keywords:** Arsenic, Skin lesions, Anemia, Ferritin, Inflammation, C-reactive protein

## Abstract

**Background:**

In the Ganges Delta, chronic arsenic poisoning is a health concern affecting millions of people who rely on groundwater as their potable water source. The prevalence of anemia is also high in this region, particularly among women. Moreover, arsenic is known to affect heme synthesis and erythrocytes and the risk of arsenic-induced skin lesions appears to differ by sex.

**Methods:**

We conducted a case-control study in 147 arsenic-exposed Bangladeshi women to assess the association between anemia and arsenic-induced skin lesions.

**Results:**

We observed that the odds of arsenic-related skin lesions were approximately three times higher among women who were anemic (hemoglobin < 120 g/L) compared to women with normal hemoglobin levels [Odds Ratio (OR) = 3.32, 95 % Confidence Intervals (CI): 1.29, 8.52] after adjusting for arsenic levels in drinking water and other covariates. Furthermore, 75 % of the women with anemia had adequate iron stores (serum ferritin ≥12 μg/L), suggesting that the majority of anemia detected in this population was unrelated to iron depletion.

**Conclusions:**

Considering the magnitude of arsenic exposure and prevalence of anemia in Bangladeshi women, additional research is warranted that identifies the causes of anemia so that effective interventions can be implemented while arsenic remediation efforts continue.

**Electronic supplementary material:**

The online version of this article (doi:10.1186/s12889-016-2824-4) contains supplementary material, which is available to authorized users.

## Background

National groundwater surveys suggest that millions of people living in Bangladesh are at risk of ingesting arsenic-contaminated water as the result of a public health initiative that switched the population’s drinking water from surface to groundwater by installing shallow tubewells [27]. The concentration of arsenic in these shallow tubewells ranges from non-detectable to upwards of 2,000 μg/L. It is estimated that approximately 59 % of the tubwells tested exceed the Bangladesh drinking water standard of 50 μg/L [27]. Inorganic arsenic is a known human carcinogen, and chronic exposure increases the risk of cancers of the skin, bladder, lung, and kidney [10, 39, 41]. Chronic exposure to arsenic is also associated with non-cancer outcomes including bronchitis, cardiovascular disease, adverse reproductive outcomes, and type 2 diabetes [12, 14, 15, 24, 33, 40]. The first dermal signs of arsenic toxicity manifest as melanosis and/or leukomelanosis, keratosis, and hyperkeratosis of the palms and soles [15]. Prospective epidemiologic studies have demonstrated that the risk of arsenic-related skin lesions increases in a dose-dependent manner and that the presence of skin lesions are highly associated with risk of cancer later in life [4, 23].

Considerable evidence supports the observation that arsenic can influence many aspects of the heme system. Previous research has shown that arsenic decreases heme metabolism and can bind to hemoglobin, resulting in lower hemoglobin concentrations [22, 25]. Arsenic has been shown to alter erythrocyte morphology and induce erythrocyte death [7, 17, 28, 48]. Arsenic also depresses bone marrow, which can lead to anemia (hemoglobin < 120 g/L in non-pregnant adults), leukopenia, and thrombocytopenia [38]. There have also been two medical case reports describing patients with megaloblastic anemia in conjunction with chronic arsenic exposure where one person had normal folate status [46] whereas the other case had low folate status [44]. Additionally, previous research by our group found that men with higher hemoglobin levels when measured continuously had lower odds of skin lesions after adjusting for confounders [9].

The prevalence of anemia in rural Bangladeshi women is high. Population-based surveys have reported that up to 43 % of rural Bangladeshi adolescent girls and 49 % of pregnant women are anemic [1]. Anemia has significant health implications for both women and their offspring because women who are anemic during pregnancy are at increased risk of maternal mortality and preterm birth [35]. Additionally, other studies have reported that women who were anemic prior to conception were more likely to have infants with lower birth weight and more fetal growth restriction [34, 35]. Iron deficiency is frequently cited as the most common cause of anemia in underprivileged populations [42]. However, one study, which examined both anemia and iron status among rural Bangladeshi women, reported that iron deficiency was absent in a large percentage of Bangladeshi women who exhibited anemia and that parity and thalassemia were the most common predictors of anemia [31]. This study, which also looked at arsenic exposure, did not report any relationship between drinking water arsenic (>50 μg/L) and anemia. However, another large population study conducted in rural Bangladesh reported that hemoglobin levels <100 g/L were significantly associated with high urinary arsenic concentrations, lower body mass index, low intake of iron, and contraceptive use [21].

Consequently, we used existing data collected in a case control study designed to examine susceptibility factors for arsenic-related skin lesions to further examine the relationship between anemia and skin lesions among Bangladeshi women. Since infection and inflammation can produce an acute-phase response that increases hepatic ferritin synthesis irrespective of iron status [8], we measured both serum ferritin and high sensitive C-reactive protein (hs-CRP), a commonly used biomarker of inflammation, to better characterize anemia and iron status in this sub-set of women. Our primary goal in this study was to evaluate the following related hypotheses: 1) women with anemia defined as hemoglobin levels <120 g/L had an increased risk of arsenic-related skin lesions, 2) women with poor iron status defined as a serum ferritin level ≤12 μg/L had an increased risk of arsenic-related skin lesions; and 3) women with inflammation defined as hs-CRP levels ≥10 mg/L had an increased risk of arsenic-related skin lesions.

## Methods

### Study population

A case-control study was performed in the Pabna district of Bangladesh from 2001 to 2003. Participant selection and study procedures have been described previously in detail [9, 30]. Briefly, 900 individuals with one or more type of arsenic-related skin lesion (diffuse/spotted melanosis, diffuse/spotted keratosis, hyperkeratosis, or leukomelanosis) and 900 controls were recruited. Controls did not have any visible sign of arsenic-related skin lesions and were matched one-to-one with cases on location of residence, age (within 3 years), and gender. All participants were ≥16 years of age and were a resident in one of the 23 villages served by Dhaka Community Hospital Trust primary care clinics. A single physician who was trained by Dhaka Community Hospital was responsible for ascertaining status of skin lesions and was blinded to arsenic exposure status in the participant’s drinking water. However, given that severe skin lesions frequently manifest on patient’s hands, it is likely that the field staff was aware of chronic arsenic exposure status in some cases. The physician also collected a toenail sample and a venous blood sample. A trained staff member administered questionnaires that collected behavioral and demographic information, including age, sex, marital status, tobacco and betel nut habits, education, drinking water history, dietary habits, etc. A water sample was collected from the participant’s home.

For this analysis, we selected all women who were age 18 to 33 years and who had sufficient quantities of archived serum available for additional biomarker analysis. Our rationale for restricting to this age range was to capture women of reproductive age. The minimum age of 18 years was due to the initial eligibility requirement in the original case control study whereas the maximum age of 33 years is a somewhat arbitrary cutoff that allowed us to include all women in this age range rather than randomly sample across a wider age range. This strategy resulted in 75 cases and 72 unmatched controls.

### Ethics statement

All study procedures were approved by the Human Subjects Committee of the Harvard School of Public Health and Dhaka Community Hospital. Additionally, the current analysis was approved by the Human Subject Committee at the University of Massachusetts Amherst. All participants provided informed consent prior to participating in any study activities.

#### Anemia and iron status assessment

At the time of enrollment, a whole blood sample was collected. Serum was isolated by centrifugation within several hours of collection and frozen at –20 °C immediately after processing. Serum samples were shipped on dry ice to Harvard School of Public Health, where samples underwent one freeze-thaw cycle in order to create aliquots prior to storage at -80 °C. Frozen serum samples were transported on dry ice to the Department of Nutrition, University of Massachusetts Amherst, where they were stored at -80 °C until biomarker assessment.

Hemoglobin was measured using Sahli’s method at the time the participant was recruited into the study [5]. Following the WHO definition, women who had a hemoglobin level <120 g/L were defined as having anemia [42].

Serum ferritin concentrations were measured using a commercially available enzyme immunoassay kit following manufacturer’s instructions (Ramco Laboratories Inc., Stafford, TX); absorbance was read at a wavelength of 490 nm with a correction filter set at 520 nm (MRX Microplate Reader, Revelation). Women who had serum ferritin levels ≤12 μg/L were defined as having iron deficiency; women who also had a hemoglobin level <120 g/L were classified as having iron-deficiency anemia.

### Inflammation assessment

High-sensitivity C-reactive protein (hs-CRP) was measured in serum using a commercially available immunoassay test kit following manufacturer’s instructions (Biocheck, Inc., Foster City, CA); absorbance was read at a wavelength of 450 nm (MRX microplate reader). This assay has been validated by numerous researchers [19, 45]. We chose to classify women with hs-CRP levels ≥10 mg/L as having inflammation. This cut-off level was chosen because it is related to levels in patients with acute inflammation, infections and any other serious inflammatory condition [29]. Because ferritin levels can be speciously elevated due to infection or inflammation, rather than iron sufficiency, we also classified women as having iron deficiency in the presence of inflammation if serum ferritin levels were <50 μg/L and hs-CRP was ≥10 mg/L.

#### Arsenic exposure assessment

Arsenic was measured in participants’ drinking water and their toenails at the time the participant enrolled in the study. Arsenic levels in toenails are used as biomarkers of personal exposure and provide an integrated measure of exposures that occurred over the previous year and are highly correlated with arsenic exposure from drinking water [26].

Drinking water samples were collected from the tubewell identified by the participant as her primary source of drinking water. The samples were preserved with nitric acid and stored at room temperature. Arsenic concentrations in the water were measured using inductively-coupled plasma mass spectrometry (ICP-MS) by Environmental Laboratory Services, North Syracuse, NY, USA. The method limit of detection was 1 μg As/L. Samples below the limit of detection were set to 0.5 μg/L. The average percentage of recovery of standard reference material (PlasmaCAL multielement QC standard #1 solution, SCP Science, Canada) was 104.6 %.

Toenail clippings underwent a cleaning process to remove external contamination before being digested in ultra-pure nitric acid following the method described by Chen et al. [11]. Arsenic concentrations were quantified using ICP-MS (ICP-MS Model 6100 DRC, Perkin-Elmer, Norwalk, CT) at the Harvard School of Public Health. The measured concentrations were batch-corrected for any detectable arsenic in method blanks, and this corrected value was subsequently used in all statistical analyses. The average percentage of recovery of standard reference materials NIST 1643 (Trace Metals in Water) and CRM hair was 92.6 % and 94.1 %, respectively.

### Statistical analysis

Descriptive statistics and correlation coefficients were calculated for all variables. Chi-square tests for categorical data, t-tests for comparison of means, and one-sided Wilcoxon rank sum test were used to 1) compare the characteristics between the subset of women included in this analysis and all other women recruited into the case control study and 2) compare characteristics between women who had arsenic-related skin lesions and women without arsenic-related skin lesions. The distribution of serum ferritin, hs-CRP, water arsenic, and toenail arsenic were highly skewed and subsequently transformed using natural logarithms to achieve normal distributions. These transformed variables were then used in the linear and logistic regression models.

We used separate linear and logistic regression models to evaluate the association between arsenic-related skin lesions (dependent variable) and *i)* anemia (anemia [yes/no] and hemoglobin [continuous]), *ii)* iron status (iron deficiency [yes/no], serum ferritin (continuous), iron deficiency with inflammation [yes/no]), *iii)* inflammation (inflammation [yes/no],and *iv)* hs-CRP (continuous). Due to the small number of women who were classified with iron-deficiency anemia, the association between this condition and skin lesions was not evaluated. Crude and adjusted odds ratios (OR) and 95 % confidence intervals (CI) were computed for the parameters of interest. The effect-estimates for the continuous variables were scaled by interquartile range (IQR), which reflects the change in the odds of skin lesions per increase in 1 IQR of hemoglobin, serum ferritin, or hs-CRP. Covariates were only included in the models if they were significantly associated with arsenic exposure, skin lesions, or considered to be confounders. Consequently, final models were adjusted for body mass index (BMI, <18.5 kg/m^2^, 18.5–24.9 kg/m^2^, and >25 kg/m^2^), age, and education. To facilitate the translation of model results, age was centered at its mean, and education was centered at primary educational level.

All statistical analyses were completed using SAS version 9.3 (Cary, North Carolina, USA).

## Results

This analysis highlighted a subset of 147 women who participated in a case-control study of skin lesions conducted from 2001–2003. To determine if we had introduced bias into our study, we compared this subset to all women recruited into the case control study (*n* = 543). We observed that the subset of women were younger (*p* < 0.001), less likely to ever have been married (*p* < 0.001), less likely to chew betel nuts (*p* < 0.001), more likely to use a tubewell that had lower arsenic levels (*p* = 0.02), and had completed more years of formal schooling (*p* < 0.001), compared to the larger group of women who were enrolled in the case control study (Table 1). Consequently, the subset of women included in this analysis may not reflect the general population of women of reproductive age in this region. Instead, this subset reflects women of reproductive age with more moderate arsenic exposure levels and a higher educational status.

**Table 1:** Selected characteristics compared between the subset of women who were eligible for this analysis and all other women recruited into the case control study

Characteristic	All women (*n = 543*)		Subset (*n = 147*)		*p* value
Variables	*n*	Mean ± SD	*n*	Mean ± SD	
Age (years)	542	34.9 ± 11.3	147	25.5 ± 5.0	<0.001
		Median ± IQR		Median ± IQR	
Water As (μg/L)	526	32.9 ± 269.4	146	17.2 ± 120.4	0.02
Toenail As (μg/g)	540	2.70 ± 6.97	145	2.12 ± 3.65	0.10
		%		%	
Skin Lesions					0.80
No	272	50.18	72	48.98	
Yes	270	49.82	75	51.02	
Body Mass Index (kg/m^2^)					0.70
<18.5	158	29.10	44	29.93	
18.5–24.9	332	61.14	92	62.59	
≥25	53	9.76	11	7.48	
Marital Status					<0.001
Not married	72	13.33	37	25.17	
Ever married	468	86.67	110	74.83	
Education					<0.001
No formal education	318	58.56	53	36.1	
Primary	74	13.63	17	11.56	
Secondary and above	150	27.62	77	52.38	
Chews betel nut					<0.001
No	385	71.2	131	89.7	
Yes	156	28.8	15	10.3	
Water Arsenic					0.16
≤10 ug/L	196	36.10	65	44.22	
10–50 ug/L	130	23.94	34	23.13	
>50 ug/L	217	39.96	48	32.65	
Hemoglobin (g/L)					0.99
Normal (Hb ≥120 g/L)	443	81.58	120	81.63	
Anemia (Hb <120 g/L)	100	18.42	27	18.37	

The median concentration of arsenic in the tubewells used by the subset of women in this study was 17.2 μg As/L (Table 1). Participants, on average, reported using their current tube well for 7.5 years (SD: 6.3 years; Range = 1–35 years). Toenail arsenic reflects cumulative exposure over the past year and the concentration of arsenic in toenails are highly correlated with arsenic concentrations in drinking water (σ_spearman_ = 0.42, *p* value <0.0001). Anemia and poor iron status defined as a serum ferritin level ≤12 μg/L were fairly common with 18.4 % (*n* = 27) of the women considered to be anemic (hemoglobin <120 g/L) and 18.4 % (*n* = 27) of the women considered to be iron depleted (serum ferritin ≤12 μg/L) (Table 2). Yet, only 25.9 % (*n* = 7) of women who were anemic were also classified as iron depleted, suggesting that iron deficiency anemia was not the predominant type of anemia in this population. We also observed that 9.5 % (*n* = 14) of the women were classified as having systemic inflammation (hs-CRP ≥ 10 mg/L), suggesting a possible acute response to infection. Because inflammation can increase serum ferritin levels, which was also observed in this study by a significant positive correlation between hs-CRP levels and serum ferritin (*p* value = 0.001), we extended the definition of iron depletion to include serum ferritin level to <50 μg/L in the presence of inflammation. This extension identified six additional women with iron depletion, which increased the estimated proportion of women with iron depletion to 22.5 % (*n* = 33).

**Table 2:** Comparison of selected characteristics between women with skin lesions and women without skin lesions

Characteristic	Controls (*n* = 72)		Cases (*n* = 75)		*p* value
Variables	*n*	Mean ± SD	*n*	Mean ± SD	
Age (years)	*72*	25.7 ± 5.3	*75*	25.4 ± 4.8	0.99 (0.93, 1.05)
Hemoglobin (g/L)	*71*	128.9 ± 10.1	*75*	123.9 ± 14.5	0.72 (0.55, 0.95)
		Median ± IQR		Median ± IQR	
Ferritin (μg/L)	*72*	27.35 ± 35.5	*75*	29.4 ± 39.7	1.14 (0.80, 1.61)
hs-CRP (mg/L)	*63*	0.71 ± 2.44	*71*	1.11 ± 2.57	1.20 (0.92, 1.55)
Water As (μg/L)	*72*	14.5 ± 48.4	*74*	37.7 ± 268.0	1.04 (0.92, 1.19)
Toenail As (μg/g)	*71*	1.58 ± 1.65	*74*	3.38 ± 8.55	1.88 (1.32, 2.68)
		%		%	
Body Mass Index (kg/m^2^)					
<18.5	*15*	20.8	*29*	38.7	3.38 (0.85, 13.41)
18.5–24.9	*50*	69.4	*42*	56.0	2.30 (1.09, 4.85)
≥25	*7*	9.7	*4*	5.3	Ref.
Marital Status					
Single/Unmarried	*17*	23.6	*20*	26.7	Ref.
Ever Married	*55*	76.4	*55*	73.3	0.85 (0.40, 1.79)
Education					
No formal education	*23*	31.9	*30*	40.0	1.74 (0.86, 3.52)
Primary	*5*	6.94	*12*	16.0	0.54 (0.17, 1.76)
Secondary and above	*44*	61.11	*33*	44.0	Ref.
Chews betel nut					
No	*64*	88.9	*67*	90.5	Ref.
Yes	*8*	11.1	*7*	9.5	1.05 (0.37, 2.96)
Water Arsenic					
≤10 ug/L	*30*	41.7	*35*	46.7	Ref.
10–50 ug/L	*24*	33.3	*10*	13.3	2.80 (1.16, 6.78)
>50 ug/L	*18*	25.0	*30*	40.0	0.70 (0.33, 1.50)_
Inflammation					
No (hs-CRP <10 mg/L)	*65*	90.3	*68*	90.7	Ref.
Yes (hs-CRP ≥10 mg/L)	*7*	9.7	*7*	9.3	1.05 (0.35, 3.15)
Iron depletion					
No (ferritin >12 μg/L)	*58*	80.6	*62*	82.7	Ref.
Yes (ferritin ≤12 μg/L)	*14*	19.4	*13*	17.3	1.15 (0.50, 2.66)
Iron depletion w/inflammation					
No	*55*	76.4	*59*	78.7	Ref.
Yes (ferritin ≤50 μg/L)	*17*	23.6	*16*	21.3	1.15 (0.50, 2.66)
Anemia					
No (hemoglobin ≥120 g/L)	*64*	88.9	*56*	74.7	Ref.
Yes (hemoglobin <120 g/L)	*8*	11.1	*19*	25.3	2.71 (1.10, 6.68)

Women with skin lesions were more likely to use a tubewell with arsenic concentrations above the Bangladesh drinking water standard of 50 μg/L compared to women without skin lesions (*p* value = 0.01), have higher toenail arsenic concentrations (*p* < 0.001), and have lower body mass indexes (*p* = 0.05) (Table 2). Women with skin lesions were also more likely to have lower hemoglobin levels (123.9 g/L versus 128.9 g/L, *p* = 0.02) and more likely to be anemic (25.3 % versus 11.1 %, *p* = 0.03). Additionally, women with skin lesions appeared to have elevated hs-CRP (1.11 mg/L versus 0.71 mg/L, *p* = 0.06).. Other characteristics, including age, serum ferritin, education, chewing betel nut, and marital status, did not differ between cases and controls (Table 2).

Separate logistic regression models examined the associations between skin lesions and anemia, iron status, and inflammation (Table 3). After adjusting for arsenic exposure, age, educational status and BMI, the odds of skin lesions were approximately 3 times higher in women who were anemic compared to women without anemia. The odds ratios between anemia and skin lesions were consistent whether arsenic exposure was measured in drinking water or toenails. This suggested that the odds of skin lesions were reduced by approximately 40 % per IQR in hemoglobin levels after adjusting for confounders (aOR_water As_: 0.60, 95 % CI: 0.39, 0.92; aOR_toenail As_: 0.58, 95 % CI: 0.37, 0.90). No significant differences in the odds of skin lesions were observed for women with inflammation compared to women without inflammation or for women with iron depletion compared to women with sufficient iron in adjusted models.

**Table 3:** Odds ratios (OR) and confidence intervals (95 % CI) of skin lesions for women exposed to arsenic (As) with anemia, inflammation, iron depletion, and iron depletion in the presence of inflammation

Reference group	Model 1^*^		Model 2^†^	
	OR	95 % CI	OR	95 % CI
Drinking water As				
Anemia				
Hemoglobin < 120 g/L	2.87	(1.16, 7.11)	3.32	(1.29, 8.52)
Hemoglobin ≥ 120 g/L	Ref.	-	Ref.	-
Inflammation				
hs-CRP ≥10 mg/L	1.10	(0.36, 3.36)	1.11	(0.35, 3.52)
hs-CRP < 10 mg/L	Ref.	-	Ref.	-
Iron depletion				
Ferritin ≤ 12 μg/L	1.20	(0.51, 2.84)	1.09	(0.44, 2.71)
Ferritin > 12 μg/L	Ref.	-	Ref.	-
Iron depletion w/inflammation				
Ferritin < 50 μg/L and hs-CRP ≥ 10 mg/L	1.19	(0.54, 2.62)	1.03	(0.44, 2.39)
Ferritin ≥ 50 μg/L and hs-CRP < 10 mg/L	Ref.	-	Ref.	-
Toenail As				
Anemia				
Hemoglobin < 120 g/L	2.86	(1.08, 7.62)	3.16	(1.17, 8.56)
Hemoglobin ≥ 120 g/L	Ref.	-	Ref.	-
Inflammation				
hs-CRP ≥10 mg/L	1.09	(0.33, 3.57)	1.03	(0.30, 3.49)
hs-CRP < 10 mg/L	Ref.	-	Ref.	-
Iron depletion				
Ferritin ≤ 12 μg/L	0.73	(0.30, 1.78)	0.74	(0.29, 1.88)
Ferritin > 12 μg/L	Ref.	-	Ref.	-
Iron depletion w/inflammation				
Ferritin < 50 μg/L and hs-CRP ≥10 mg/L	0.77	(0.36, 1.77)	0.72	(0.30, 1.75)
Ferritin ≥ 50 μg/L and hs-CRP <10 mg/L	Ref.	-	Ref.	-

^*^Model 1 only adjusts for arsenic exposure

^†^Model 2 adjusts for arsenic exposure, age, educational status, and body mass index

We further evaluated the dose-response relationship between skin lesions and tertiles of hemoglobin, hs-CRP, and serum ferritin using separate logistic regression models that adjusted for arsenic exposure (either in drinking water or toenails), age, educational status, and BMI (Table 4). This approach also suggested that women with higher levels of hemoglobin had a reduced risk of skin lesions compared to women with the lowest levels of hemoglobin in models that adjusted for arsenic in drinking water or toenails but the trend was not significant (drinking water As, *p* = 0.18; toenail As, *p* = 0.20). This suggested that the clinical cut-off level for hemoglobin-related anemia reasonably characterized the risk between this factor and skin lesions. We also observed a positive trend between women with the higher levels of hs-CRP and increased odds of skin lesions (drinking water As, *p* = 0.05; toenail As, *p* = 0.08) compared to women with the lowest levels of hs-CRP (aOR _water As_ = 2.32, 95 % CI: 1.00, 5.37; aOR_toenail As_ = 2.03, 95 % CI: 0.85, 4.87). This suggested an association between inflammation and skin lesions. No apparent trend was observed between the odds of skin lesions and serum ferritin tertiles.

**Table 4:** Comparisons of the odds ratios (OR) and confidence intervals (95 % CI) for skin lesions in women by tertiles of hemoglobin, hs-CRP, and serum ferritin

Outcome	Drinking water as^*^		Toenail as^†^	
	Adjusted OR	95 % CI	Adjusted OR	95 % CI
Hemoglobin (g/L)				
High (>130)	Ref	-	Ref.	-
Middle (120–130)	1.32	0.55, 3.20	1.32‡	0.54, 3.26
Low (<120)	2.21	0.96, 5.13	2.18‡	0.90, 5.27
*P for trend*		*0.16*		*0.20*
hs-CRP (mg/L)				
High (>1.90)	2.32	(1.00, 5.37)	2.03	0.85, 4.87
Middle (0.57–.90)	2.47	(1.08, 5.65)	2.58	1.06, 6.10
Low (<0.57)	Ref	-	Ref	-
*P for trend*		*0.05*		*0.08*
Serum ferritin (μg/L)				
High (>41.3)	1.35	(0.57, 3.19)	0.99	0.40, 2.46
Middle (20.6–41.3)	1.37	(0.59, 3.20)	1.07	0.44, 2.61
Low (<20.6)	Ref	-	Ref	-
*P for trend*		*0.72*		*0.98*

^*^Adjusted for arsenic exposure in drinking water, age, educational status, and BMI

^†^Adjusted for arsenic exposure in toenails, age, educational status, and BMI

‡Adjusted for arsenic exposure in toenails, age, and BMI

## Discussion

The results from this population-based case control study show that Bangladeshi women of reproductive age with anemia had a 3-fold increased odds of skin lesions compared to women who were not anemic, after adjusting for arsenic exposure and other covariates. Furthermore, only 25.9 % of the women with anemia were iron deficient, which suggests that the majority of anemia in this population was not related to iron status. Another study conducted in rural Bangladeshi women also reports that the majority of anemia observed was not due to iron deficiency but rather to parity and thalassemia [31], two risk factors that were not captured in our study. However, the women included in this analysis were younger than the average rural Bangladeshi woman of reproductive age and may not have had as many children as observed by Merrill et al.

Considering the public health significance of anemia on maternal and child health, as well as the magnitude of arsenic-related illness in this region, further research is needed to identify the causes of anemia in this population to develop appropriate interventions. For instance, inadequate intakes of folate or vitamin B_12_ may be contributing to the anemia observed in this population. These micronutrients are also required to metabolize inorganic arsenic [20]. Several studies have shown that deficiencies in these nutrients increase the risk of arsenic-related health effects in both humans and animals [16, 32, 43]. Additionally, the prevalence of hyperhomocysteinemia is reported to be high in this area [20], thus supporting the notion that B-vitamin status and folate status in particular may be compromised in this population. If this were the case, it would result in megaloblastic anemia rather than the microcytic anemia associated with iron-deficiency [2]. Histological examination of the blood would be able to discern between these two types of anemia and would provide important treatment information since megaloblastic anemia results from folate and vitamin B12 deficiency, whereas microcytic anemia results from iron deficiency. Interestingly, megaloblastic anemia has been observed in patients with low folate status and adequate folate status who were suffering from chronic arsenic poisoning [44, 46]. Thus, further data describing the relationship between chronic arsenic exposure, specific types of anemia, and nutritional status could help gain better insights into the pathology of arsenic toxicity and potential treatments.

It is also possible that arsenic exerts a direct toxic effect on erythrocytes that is contributing to the observed anemia. Several studies have shown that arsenic alters heme metabolism and contributes to lower hemoglobin concentrations [7, 17, 22, 28, 38, 48]. While our study did not observe any consistently significant association between anemia or hemoglobin levels and arsenic exposures (see Additional file 1: Table S1), another large population-based study conducted in an arsenic-endemic area of Bangladesh reported that high levels of urinary arsenic were associated with low hemoglobin levels (<100 g/L) in both men and women [21]. The lack of consistent association between anemia and arsenic in our sample could be a function of its smaller population size.

We also suggest a positive association between hs-CRP and skin lesions with the strongest association observed among the third of the participants with the highest levels of this inflammatory biomarker. It is plausible that chronic arsenic exposure produces systemic inflammation, which may be an underlying mechanism in the pathogenesis of skin lesions. Several *in vivo* studies have shown than arsenic can induce hs-CRP production [18], increase vascular inflammation by inhibiting endothelial nitric oxide synthase [13, 36, 37], and induce erythrocyte death by increasing cytosolic Ca(2+) [28], which in turn can have pro-inflammatory effects [6]. Thus, there may be a cycle through which arsenic stimulates biochemical pathways that generate oxidative stress, which negatively impacts hematopoiesis as well as the underlying mechanism of skin diseases. However, since our case-control study was cross-sectional, it is not possible to determine the temporality of the relationship between arsenic, hematopoeisis, inflammation, and skin lesions. Also, due to the small sample size we could not look at interactions between these parameters.

The characteristics for the women included in this group were somewhat different than the larger female population recruited into the skin-lesion study, and thus our results might not be generalizable to women of all ages who reside in arsenic-endemic regions. For instance, the prevalence of chewing betel nut was lower in this subset of women than the wider population which is relevant because betel nut chewing is a risk factor for folate deficiency in pregnant women [23]. Also, the prevalence of anemia in our population was 18.4 %, which was lower than what has been observed in other female, non-pregnant populations in Bangladesh [1]. This lower percentage could be due the selected subpopulation of women included in this analysis or the use of the Sahli’s method to measure hemoglobin in the field which is a colorimetric method that was used because of its simplicity and ability to deliver results immediately to participants. This method can also be subject to error because it relies on a visual comparison to determine hemoglobin concentrations. However, a study which compared the Sahli’s method to autoanalyzer methods reports that the Sahli’s method was in good agreement with the autoanalyzer with a sensitivity of 98.2 % and specificity of 66.2 % [3]. Also, the technicians that performed the Sahli’s method were blinded to the participant’s drinking water arsenic levels, which would minimize bias. However, since severe arsenic poisoning manifests frequently manifests itself as palmar skin lesions, it is possible that field staff were aware of arsenic status in some cases which could introduce systematic misclassification of hemoglobin levels for patients with palmar lesions. On a similar note, we classified as not anemic 27 subjects whose hemoglobin concentration was exactly 120 g/L. If we included these individuals in the anemic group, the estimated prevalence of anemia would have increased to 36 %.

Another limitation that must always be recognized is potential misclassification of arsenic exposure, which would introduce additional variability into the model. However, we minimize this possibility by using personal measurements of current exposure (drinking water arsenic) and historical measurements of cumulative exposure (toenails). Another limitation is that we did not collect information about genetic risk factors for anemia, such as Thallasemia, or additional blood parameters such as mean cell volume (MCV) or serum levels of folate and vitamin B_12,_ which would have allowed us to distinguish between different causes of anemia. Finally, it would have been ideal to include assessment of soluble transferrin receptor concentration since this biomarker reflects iron deficiency and is not affected by infection or inflammation. However, we had limited biospecimens and needed to prioritize assays. Thus, we chose to measure hs-CRP to assess inflammation even though serum hs-CRP level spikes faster than ferritin and has a shorter half-life [47]. As a result, we may not have been able to identify all women who were actually iron deficient despite “normal” ferritin levels {49]. Histological examination of the blood sample was not performed which would have identified the presence of microcytic and megaloblastic anemia nor were folate or vitamin B12 levels measured in these participants due to limited serum availability. Future studies should consider including this information in order to further characterize anemia in this population and identify appropriate treatment strategies.

## Conclusion

In conclusion, this case-control study identified anemia and inflammation as significant risk factors for skin lesions after controlling for arsenic exposure and other confounders. Further studies are thereby justified to determine the causes or types of anemia in this population, to explain the causal relationship between anemia and skin lesions, and to better define the role that arsenic plays in both these conditions so that appropriate public health interventions can be implemented.

## Consent

Informed consent was obtained from all participants prior to their participation in this study. A copy of the written consent is available for review by the Editor of this journal.

## Additional file

Additional file 1: Table S1. Comparisons of the odds ratios (OR) and confidence intervals (95 % CI) for anemia in women by arsenic exposure. (DOCX 11 kb)

## Abbreviations

As: arsenic
aOR: adjusted Odds Ratio
BMI: body mass index
CI: confidence interval
hs-CRP: high sensitive C-reactive protein
IQR: interquartile range
OR: odds ratio
p: *p* value
