# Supplementary material for: A cross sectional study of anemia and iron deficiency as risk factors for arsenic-induced skin lesions in Bangladeshi women
Source: BMC Public Health. 2016 Feb 16;16:158. doi: 10.1186/s12889-016-2824-4 (PMC4754934; doi:10.1186/s12889-016-2824-4)
Supplement: Additional file 1: Table S1. — Comparisons of the odds ratios (OR) and confidence intervals (95 % CI) for anemia in women by arsenic exposure. (DOCX 11 kb) [file 12889_2016_2824_MOESM1_ESM.docx]

**Additional file 1: Table S1.** Comparisons of the odds ratios (OR) and confidence intervals (95% CI) for anemia in women by arsenic exposure.

| **Outcome** | **Drinking Water As** | | **Toenail As** | |
| --- | --- | --- | --- | --- |
|  | OR | 95% CI | OR | 95% CI |
| ***All women (n = 543)*** |  |  |  |  |
| Anemia |  |  |  |  |
| Yes | 0.96 | 0.88, 1.04 | 1.20 | 1.20, 1.41 |
| No | Ref |  | Ref |  |
| ***Subset women (n = 147)*** |  |  |  |  |
| Anemia |  |  |  |  |
| Yes | 0.92 | 0.78, 1.08 | 1.06 | 0.72, 1.57 |
| No | Ref |  |  |  |
